# Recent Advances of VO_2_ in Sensors and Actuators

**DOI:** 10.3390/nano14070582

**Published:** 2024-03-27

**Authors:** Mahmoud Darwish, Yana Zhabura, László Pohl

**Affiliations:** 1 Department of Electron Devices, Faculty of Electrical Engineering and Informatics, Budapest University of Technology and Economics, 1111 Budapest, Hungary; 2 Department of Electrical Engineering and Automation, School of Electrical Engineering, Aalto University, 02150 Espoo, Finland; yana.zhabura@aalto.fi

**Keywords:** vanadium dioxide, insulator–metal transition, sensor, strain, temperature, microactuator

## Abstract

Vanadium dioxide (VO_2_) stands out for its versatility in numerous applications, thanks to its unique reversible insulator-to-metal phase transition. This transition can be initiated by various stimuli, leading to significant alterations in the material’s characteristics, including its resistivity and optical properties. As the interest in the material is growing year by year, the purpose of this review is to explore the trends and current state of progress on some of the applications proposed for VO_2_ in the field of sensors and actuators using literature review methods. Some key applications identified are resistive sensors such as strain, temperature, light, gas concentration, and thermal fluid flow sensors for microfluidics and mechanical microactuators. Several critical challenges have been recognized in the field, including the expanded investigation of VO_2_-based applications across multiple domains, exploring various methods to enhance device performance such as modifying the phase transition temperature, advancing the fabrication techniques for VO_2_ structures, and developing innovative modelling approaches. Current research in the field shows a variety of different sensors, actuators, and material combinations, leading to different sensor and actuator performance input ranges and output sensitivities.

## 1. Introduction

Vanadium dioxide (VO_2_) is a material that undergoes a reversible transition between metal and insulator states. Insulator–metal transition (IMT) occurs at a reasonably convenient temperature and can be further modified using doping. This property of VO_2_ was first discovered in 1959 [1] and has been the subject of ongoing research endeavors ever since. Areas of interest include theoretical research in transition mechanisms, possible methods for the synthesis of VO_2_ structures, and prospective applications. Apart from the change in temperature [2,3], the transition can also be triggered by strain [4,5], pressure [6,7], light [8,9], and electrostatic fields [10,11,12]. This, combined with relatively easy synthesis, makes it even more appealing for a wider spectrum of applications.

For stochiometric VO_2_ at ambient pressure, the IMT occurs around the temperature of 340 K (67 °C), referred to as the Néel temperature [13,14,15]. The IMT of VO_2_ is a first-order transition, meaning that the transition is characterized by an abrupt and discontinuous change in properties at the transition point, as opposed to gradual, continuous changes seen in second-order phase transitions [16,17]. The IMT is coupled with a structural phase transition between high-temperature rutile VO_2_(R) and low-temperature polymorph VO_2_(M) [18,19]. This transition leads to mechanical strain as well as changes in physical properties, such as electrical and thermal conductivity, magnetic properties, and optical transmittance and absorption [20]. The electrical conductivity change is not solely caused by the phase change, as many transition metal dioxides have a similar structure and undergo a similar phase transition; however, VO_2_ and NbO_2_ undergo the IMT while compounds like MoO_2_, RuO_2_, IrO_2_, and PtO_2_ stay metallic at all temperatures [21].

### 1.1. Description of Known VO_2_ Phases

**Figure 1:**
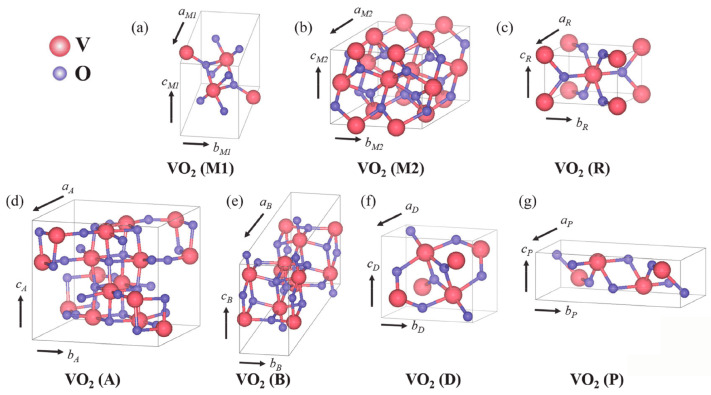
Crystal structures of VO_2_ polymorphs (copied from [22]): (**a**) VO_2_(M1), (**b**) VO_2_(M2), (**c**) VO_2_(R), (**d**) VO_2_(A), (**e**) VO_2_(B), (**f**) VO_2_(D), and (**g**) VO_2_(P) (reproduced from [22], MDPI open access CC BY 4.0 license, 2021).

Vanadium dioxide exhibits several polymorphs, each characterized by a distinct crystallographic structure and associated physical properties. Transitions between these polymorphs occur through reversible and irreversible phase transformations. The phases that have been reported so far include VO_2_(A), VO_2_(B), VO_2_(D), VO_2_(R), VO_2_(M1), VO_2_(M2), and VO_2_(P) [22]. The crystal structures of the polymorphs are shown in Figure 1.

VO_2_(R) is the high-temperature rutile polymorph that exhibits metallic properties and takes part in the IMT [22]. VO_2_(M) is the most researched low-temperature polymorph that acts as an insulator. The difference between the VO_2_(M1) and VO_2_(M2) phases is the arrangement of V atoms along the c-axis. VO_2_(M2) can be seen as an intermediate phase of the phase transition from VO_2_(M1) to VO_2_(R); however, it can be stabilized by introducing doping and high uniaxial pressure [23]. Thus, while both VO_2_(M1) and VO_2_(M2) transform into VO_2_(R) with heating above 67 °C, the VO_2_(M1) to VO_2_(R) transition is reversible.

The VO_2_(A) polymorph can take two crystal structures, low-temperature-phase VO_2_(A_L_) and high-temperature-phase VO_2_(A_H_), between which a reversible transition exists [24]. Similarly, VO_2_(M) is also an insulator polymorph exhibiting IMT behavior, and some similar applications have been proposed [25], but the first-order transition to VO_2_(R) happens at a higher temperature of around 162 °C, making it less attractive for room-temperature applications. VO_2_(B) is a metastable phase that has a layered structure, making it interesting for applications in supercapacitors and batteries [26]. It is also interesting as a step in the synthesis of VO_2_(R), to which it transitions through annealing, but at a higher temperature [27].

Another polymorph that can be used to obtain VO_2_(R), at a lower temperature compared to VO_2_(B), is VO_2_(D). It also exhibits magnetic and semiconductive properties [28]. VO_2_(P) is a conductive polymorph that is considered for applications in batteries [29] and can be transformed to VO_2_(M) by rapid annealing [22]. Other intermediate phases, including VO_2_(T), VO_2_(M3), and VO_2_(M4), some of which can be stabilized with doping and strain, have been shown in the literature [30,31].

### 1.2. Physical Characteristics of VO_2_ Phases

Due to the significant change in properties between the VO_2_(R) and VO_2_(M) polymorphs and the reversible, relatively low-temperature transition between the phases, they attract the most interest regarding potential applications. Some of the physical properties of the phases are presented in Table 1, based on [32,33,34].

It is worth noting that while the increase in heat capacity that corresponds to phase change and magnetic susceptibility value change happens at 340 K, the resistivity value change exhibits a hysteresis behavior, where the transition happens at 340 to 325 K when cooling and 335 to 350 K when heating [1,35].

### 1.3. Possible Mechanisms for the Insulator–Metal Transition

The mechanics of the IMT has been extensively studied, but a comprehensive picture that would explain the underlying mechanisms for the many phenomena observed within VO_2_ has not been formed yet. The debate primarily revolves around two competing scenarios: Peierls-like or Mott-like transition. Recent theoretical approaches have suggested a potential resolution to the debate, introducing an intermediate perspective known as “Mott-assisted Peierls” or “Mott–Peierls” [36].

A Peierls transition is a phenomenon in which a material undergoes a transition from being a metal to an insulator, caused by a lattice distortion or structural instability in the crystal lattice, with a periodic lattice deformation causing an energy gap [37]. A Mott transition, on the other hand, is triggered by changes in electron density, which can be achievable through pressure or temperature changes when the material is near a critical electron density. The transition arises from the interaction between the Coulomb forces between the electrons and their band width. Due to electron screening, the band width of the electrons is reduced, leading to unbound states that cause the material to behave like a metal at high electron densities [37].

Some studies have supported the VO_2_ IMT being well described through the Mott-like mechanism. In [38], the Mott transition was supported through the use of infrared spectroscopy. Mott-like transition was also supported through the use of a Raman experiment and an extended Brinkman–Rice picture [39], which extended to Mott transition theory, giving a more detailed explanation of the transition mechanism [40]. 

Other studies have used the Peierls-like mechanism to describe the behavior of VO_2_. Using theoretical calculations, VO_2_ was classified as a Peierls-type insulator in [41], though it was mentioned that there were reasons to consider it a Mott insulator too. An extension of the Peierls approach was used in [42] to calculate the optical properties of VO_2_, with the calculation agreeing with experimentally measured optical conductivity, along with other studies supporting the Peierls-like mechanism [43,44]. 

Many studies propose a more nuanced approach that includes both mechanisms in an attempt to more accurately describe the cause of the transition. In [45], it was shown that while the transition in VO_2_ is caused by both the Mott and Peierls effects, introducing hole doping decouples the states, suggesting further research is needed into the stability of different VO_2_ phases and the complex interaction between the transition scenarios. Similarly, Ref. [46] explored the influence of doping on VO_2_, showing either Peierls-like or Mott-like behavior.

The categorization of transitions into “Peierls” or “Mott” was questioned in [47] as an oversimplification. Electrically induced transition and doping experiments have been used to propose that for VO_2_, the Mott transition should be viewed as a “trigger mechanism” that causes a Peierls-like structural transition. Similar conclusions were reached in [48] and [49], with the authors calling the transition a Mott–Peierls transition. The role of the orbital structure of the vanadium ions in the oxide was discussed in [50] and [51], and the transition was described as a “collaborative” Mott–Peierls transition. The effect of doping on the prevalence of the Mott mechanism in the transition was described through the orbital occupancy of vanadium in [52]. Other studies have also been completed supporting the compound approach [53,54]. 

So, while a comprehensive picture has not been formed yet, the current research points towards the IMT of VO_2_ not exclusively being described as a Mott or a Peierls transition, but rather being caused by the lattice deformation in a Peierls-like mechanism that is triggered by a Mott-like change in electron density, which is heavily influenced by doping.

### 1.4. Publication Trends

As the interest in the material is growing year by year, the purpose of this review is to explore the trends and current state of progress on some of the applications proposed for VO_2_ in the field of sensors and microactuators. 

**Figure 2:**
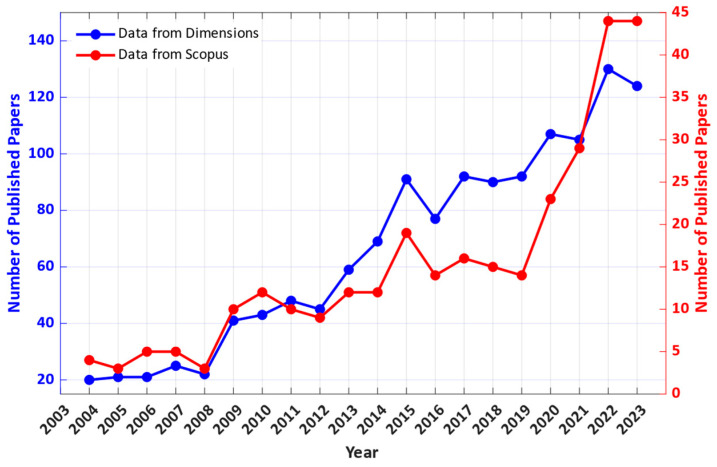
Number of published papers per year in the last 20 years that relate to vanadium dioxide and microelectronics.

**Figure 3:**
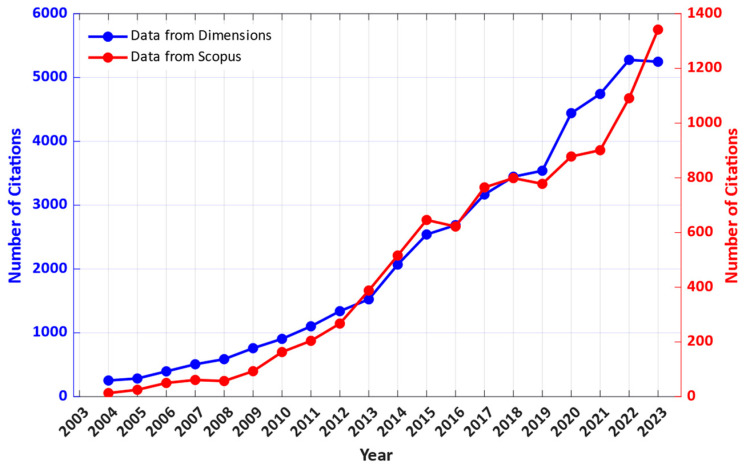
Number of citations per year in the last 20 years that relate to vanadium dioxide and microelectronics.

In the past 20 years, the cumulated number of published papers on the topic has grown to 303 from a search on Scopus and 1322 from Dimensions. To be able to analyze the trend in the number of published papers per year, it was plotted against time, for the last 20 years until 2023, excluding year 2024 as the year is still ongoing (Figure 2). Both the data from Scopus and Dimensions show a rapid growth in interest in the field, especially since around 2008. Similar trends can be seen in the number of citations, as shown in Figure 3. Table 2 presents five of the most cited papers that fall under the searched keywords.

### 1.5. Recent Reviews

Review papers provide a good basis for further research results related to vanadium oxides. The mechanism of its phase change and the physics of its operation are presented in [36,58,59]. Good overviews of the material structure and properties are given in [20,60,61,62,63]. Research on the synthesis of vanadium oxides, deposition methods, fabrication methods, and thin film fabrication are described in [59,60,63,64,65,66,67,68]. The growth of vanadium dioxide nanostructures and nanoparticle synthesis are discussed in [12,22,69]. Improvement in the optical parameters of VO_2_ is presented in [69]. 

Articles describing the practical applications of VO_2_ fall into two categories. Some of the reviews try to cover as many application areas as possible [20,36,59,60,63,66,70,71]. The other type of articles covers one or two application areas in more detail. From [61], we learned about VO_2_-based electrode design, from [67,69] about coatings, from [72] about applications in photonics, and from [12] about optical sensors. VO_2_ applications in electronics, optics, and sensors are described in detail in [22]. The research in [68] deals with gas sensors and vapour sensors.

This review paper mainly focuses on recent research results on vanadium dioxide-based actuators and sensors.

## 2. Applications: Overview

This section presents some key applications of VO_2_ in the field of sensors and actuators. It focuses on some of the more developed fields of application, which include three sensor types and one microactuator type.

### 2.1. Resistive Strain Sensor

A resistive strain sensor is a sensor that transforms a mechanical input signal, such as strain, into an electrical signal, in this case, change in resistance. Strain is expressed in a percentage, that is, the difference in length of the sample as a percentage of its length in an unstressed state. Conventionally, a positive strain value corresponds to a sample that is being stretched, i.e., under tensile strain, and a negative value corresponds to compression of the sample.

**Figure 4:**
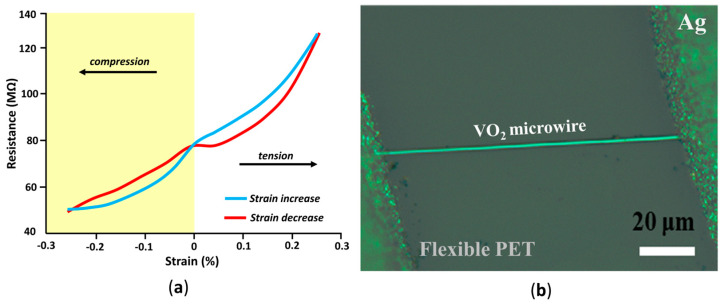
(**a**) Device resistance as a function of applied strain, showing hysteresis behavior (reproduced with permission from [73], John Wiley and Sons, 2010), (**b**) micrograph of a microwire on a flexible substrate strain sensor (reproduced with permission from Ref. [74], Royal Society of Chemistry, 2013).

In [73], VO_2_ nanobeams were grown using physical vapor deposition on a Si wafer. A nanobeam was transferred to a flexible polystyrene substrate and silver paste was used as electrodes. The device was tested under a strain of −0.25% to 0.25% and showed a relatively linear dependence between resistance and strain, as well as some hysteresis behavior, as shown in Figure 4a. The device is proposed as a sensor to quantify small strain.

Chemical vapor deposition was used in [74] to deposit VO_2_ microwires on a SiO_2_/Si substrate. Then, the microwire was transferred onto a polyethylene terephthalate (PET) substrate, and silver paint was used as electrodes. The device can be seen in Figure 4b. It was tested under a strain of −0.6% to 0.8%, with the resistance changing relatively linearly as a function of strain. The device is proposed as a flexible strain sensor, in particular, for advanced stress detectors and intelligent bionic devices.

In [75], RF sputtering was used to grow thin films of VO_2_ on a flexible glass substrate. The device resistance under applied tensile strain changed from around 20 kΩ to around 5 MΩ. The device is proposed for applications in keyboards, pressure-sensitive displays, and touch screens. Polymer-assisted deposition was used in [76] to grow a VO_2_ thin film on a SiO_2_/Si substrate, and then, the film was etched into a designed pattern and transferred between polydimethylsiloxane (PDMS) and PET films, attached to Au electrodes. The sensor is a dual-parameter sensor used to monitor a person’s real-time pulse and body temperature. The signals are separated due to different frequency characteristics.

A similar dual-parameter approach was explored in [77], where an ink was manufactured by combining VO_2_ nanobelt powder with a solution of latex, and deionized water and conductive patterns were drawn onto the substrate using a pen. The flexible sensors were used for speaking, pulse, joint bending, facial expression, hand gesture, and muscle state recognition.

### 2.2. Resistive Temperature Sensor

A resistive temperature sensor is a sensor that transforms a thermal input signal into an electrical signal, in this case, change in resistance. The temperature coefficient of resistance is defined as the slope of the linear fit for the resistance and temperature dependence. Two dual-parameter resistive temperature sensors presented in [76,77] were covered in the previous sub-section. 

Another approach to fabricating a flexible resistive temperature sensor was explored in [78]. It was made by first preparing a poly(styrene–block–butadiene–block–styrene) fabric substrate by electrospinning and pre-treating it with plasma to make it hydrophilic. Then, ink was prepared by mixing vanadium dioxide (VO_2_) nanoparticles and the aqueous solution of poly(3,4-ethylenedioxythiophene):poly(styrenesulfonate) in deionized water. Then, the VO_2_ film was spray printed onto the fabric, Ag electrodes were manufactured using an e-beam, and lastly, chemical vapor deposition was used to cover the whole area with an encapsulating layer of parylene.

The sensor was tested in temperatures of 25 °C to 45 °C, where it showed good sensitivity and linearity, as well as a relatively small hysteresis window. While the range of temperatures tested was quite narrow, the sensor is intended for use in soft AI wearables and is mainly meant to measure the temperature of human skin.

A thermal sensor intended for environmental temperature measurements, including applications in agriculture, was developed in [79]. The device was manufactured on a SiO_2_/Si wafer and consisted of Pt/Ti electrodes and a DC sputtered and annealed thin film of VO_2_. The sensor was tested under a variety of temperatures from −100 °C to 100 °C and humidities of 10% to 90% relative humidity. 

**Figure 5:**
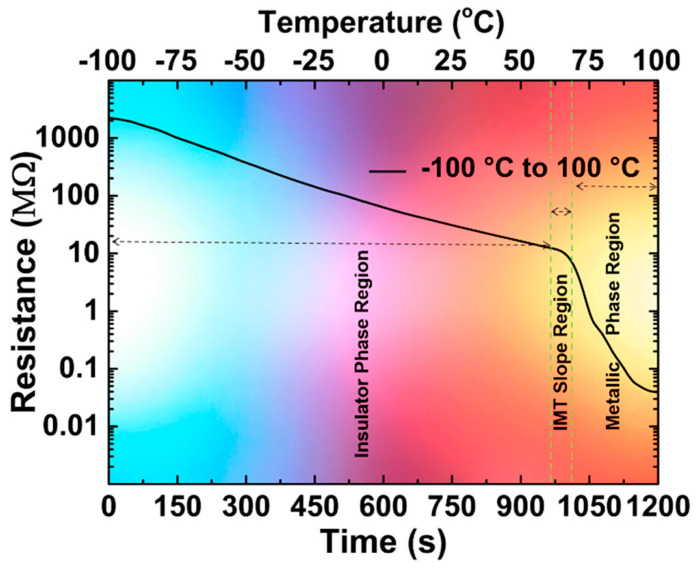
Device resistance as a function of temperature (reproduced with permission from Ref. [79], American Chemical Society, 2022).

Figure 5 shows the device performance at ambient humidity for the whole range of tested temperatures. The temperature range is divided into three areas, an insulator region (temperatures below IMT), an IMT slope region (62 °C to 68 °C), and a metallic region (temperatures above IMT), with the sensor exhibiting different behaviors in these temperature ranges.

**Figure 6:**
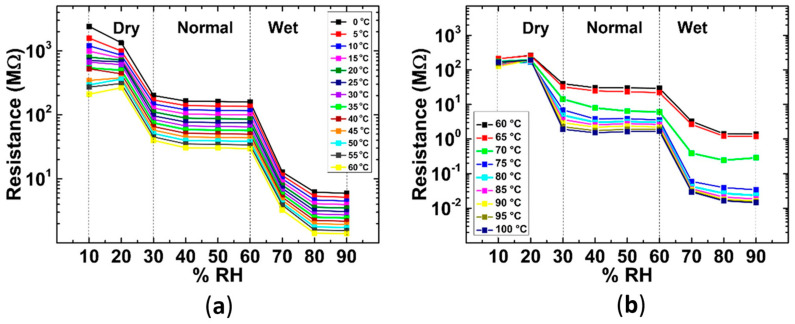
Device resistance as a function of relative humidity at different temperatures: (**a**) below transition temperature, (**b**) above transition temperature (reproduced with permission from Ref. [79], American Chemical Society, 2022).

The study also takes into account the influence of humidity on the sensor’s performance. It presents a theoretical explanation for the influence of humidity from a density functional theory standpoint. Figure 6 presents the dependence of the device resistance on relative humidity, from 10% to 90%. In the metallic phase region, Figure 6a, the change in resistance due to the change in humidity seems to be mostly independent from the temperature. However, in the higher temperature region, Figure 6b, the nature of resistance change due to humidity depends on the temperature. 

The humidity range can be divided into dry, normal, and wet conditions, in which the behavior of the sensor is relatively stable with changes in humidity. Overall, with increasing humidity, the resistance decreased, while keeping the overall temperature dependence. The sensor can be used for temperature sensing under any humidity conditions with some digital processing of the signal or additional packaging.

### 2.3. Mechanical Microactuators

A mechanical microactuator is an actuator that transforms an electrical input signal of some power, measured in Watts, to a mechanical output signal, expressed as deformation or displacement, as measured in the percentage of a respective size or µm, respectively.

One study [80] proposes a vertical stack cantilever microactuator structure. To fabricate it, first, physical vapor deposition was used to deposit Si_3_N_4_ on a Si substrate. Then, ITO was deposited as the bottom electrode via e-beam evaporation. A layer of VO_2_ was sputtered on top via RF magnetron sputtering and patterned using reactive ion etching. The top Pt electrode was deposited on top using e-beam evaporation. Then, the ITO and Si_3_N_4_ layers were reactive iron etched to open channels for substrate release through XeF_2_ etching.

Actuator deformation occurs at the phase transition of VO_2_, caused by global heating or electrically induced heating. The observed tip displacement for a cantilever length of 35 µm was 2 µm under global heating and 0.22 µm under electrically induced heating. This order of magnitude difference can be explained due to the uneven heating of the structure under electrically induced heating.

The study also reported electrical measurements, including the structure’s total resistance change due to applied voltage, observing a significant hysteresis effect, where the phase transition was observed at 1.95 V when increasing the voltage and 1.05 V when decreasing it. The study authors also proposed an electrical resistance model of the cantilever and performed a finite element modelling (FEM) simulation, which predicted a greater tip displacement. The difference was possibly attributed to the heat-induced stress being partially released through defects and grain boundaries in the VO_2_ layer as opposed to producing strain. 

Another stacked cantilever structure was explored in [81]. To fabricate the actuator, a layer of SiO_2_ was deposited by plasma-enhanced chemical vapor deposition on a Si substrate. Then, an adhesion layer of Ti and Pt electrodes were deposited by evaporation and patterned by lift-off. A sacrificial layer of Si was deposited on top via low-pressure chemical vapor deposition and patterned by reactive ion etching. Then, a SiO_2_ layer was deposited on top via low-pressure chemical vapor deposition and patterned using plasma etching. The second Ti/Pt metal layer forming an integrated resistive heater was sputtered and pattered via lift-off. Then, a layer of SiO_2_ was deposited on top and the structures were released using XeF_2_ after dicing. Finally, a VO_2_ thin film was deposited with pulsed laser deposition using a shadow mask.

The displacement versus actuation current characteristic was studied in two environments: in air and in a vacuum. The electrical power required to achieve full actuation was reported to be 16 times greater in air than in the vacuum. The tip displacement was not 0 at 0 current applied because it was measured from a flat state and there was some residual deformation caused by the fabrication process. FEM simulation was used to simulate the tip displacement.

**Figure 7:**
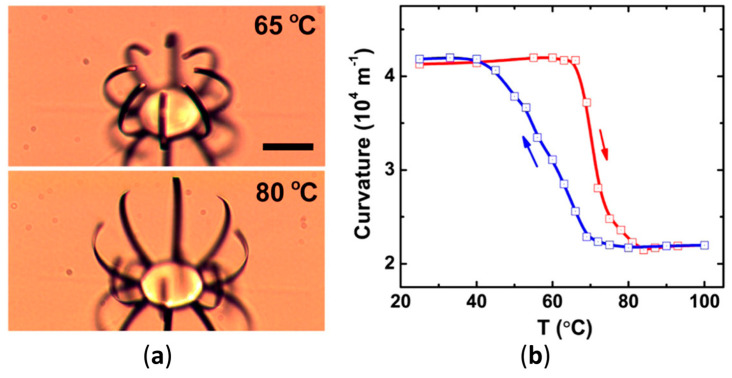
(**a**) Device shape before the phase transition (top, at 65 °C) and after (bottom, 80 °C), (**b**) curvature of the actuator as a function of its temperature (reproduced with permission from Ref. [82], American Chemical Society, 2012).

Another approach to VO_2_ microactuators is proposed in [82]. The actuator was manufactured by first depositing VO_2_ on a SiO_2_/Si substrate using pulsed laser deposition. Then, a Cr layer was deposited with e-beam evaporation and lithographically patterned. Then a photoresistant pattern was defined to protect the fixed ends of future cantilevers, and the exposed VO_2_ was reactive ion etched and finally released by buffered oxide etching. At room temperature, the released structures curved towards the Cr layer and the curvature decreased with the phase transition of VO_2_, as shown in Figure 7a.

Two actuation methods were proposed for the actuator: electrical or light actuation. The tip displacement observed was 36 µm for a 60 µm long structure. In the study, both displacement and curvature values were considered, due to curvature being a more intuitive measurement for this type of actuator. Figure 7b shows the curvature dependence from temperature.

When the device was actuated via laser, the required power was around 4 mW. Depending on where the laser was focused, either the whole palm or individual fingers could be actuated. When the device was actuated electrically, a 1.4 V voltage was applied, with 1.6 mW of electrical power causing maximum displacement.

Direct laser writing was used in [83] to produce a microactuator. To achieve this, a VO_2_ film was laser patterned, then the achieved microbelt structure was detached from the film using a tungsten probe and transferred to a SiO_2_ substrate. It was fixed using silver paste on one end and coated with a Cr layer. FEM simulation was used to determine the optimal thickness of the Cr layer.

**Figure 8:**
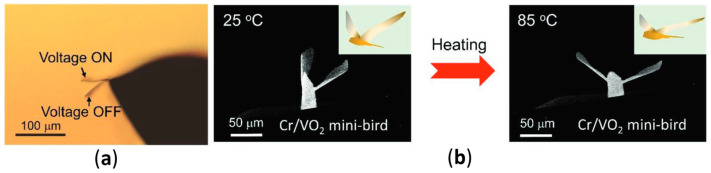
(**a**) Microbelt device in deformed and undeformed states, (**b**) bird-shaped microactuator (reproduced with permission from [83]. John Wiley and Sons, 2022).

To activate the actuator, the authors used two approaches: light and electrical. To use light for actuation, a laser with a wavelength of 808 nm was used to cause the VO_2_ heating, with a 250 mW laser causing a 25 µm tip displacement for a 40 µm cantilever. By attaching the microcantilever to a microchip with Au electrodes, the actuator could be activated with electrical current, resulting in 30 µm deflection at 120 mW electrical power applied, and the cantilever deformation can be seen in Figure 8a.

To show the possibilities of direct laser writing for microactuator fabrication, a bird-shaped actuator was manufactured, as shown in Figure 8b. The actuator was actuated by temperature via a heating stage, changing the temperature from 25 °C to 85 °C.

A more sophisticated approach to actuator control was used in [84] by incorporating a self-sensing electrode into the structure and employing a PID controller. The device was fabricated by first depositing a layer of SiO_2_ on a Si wafer using low-temperature oxide. Then, a layer of Ti and Pt were deposited using thermal evaporation and patterned with lift-off. A second SiO_2_ layer was deposited on top and patterned using deep reactive ion etching. Then, the cantilever was released using XeF_2_, and finally, a layer of VO_2_ was deposited using pulsed laser deposition.

Electrical current up to 5 mA was applied to achieve deflection of up to 100 µm for a cantilever of 550 µm in length. The resistance of the device was measured using the self-sensing electrodes, and a Boltzmann function was used to approximate the actual deflection from the resistance measurement; this approximation was then used as a feedback signal for a PID controller to adjust the driving current.

## 3. Comprehensive Review

This section presents a comprehensive review of the proposed applications for VO_2_ in the field of sensors and actuators, which inherently led to some applications being excluded due to their limited relation to the field of focus of the review. Some of the notable applications that were excluded from the review include the following:

- thermochromic films for smart window applications [85];
- thermal camouflage [86];
- radiators [87];
- applications as electrodes for batteries [88];
- applications as electrodes for supercapacitors [89];
- optical temperature sensors [90,91];
- electrical switches [92,93];
- field–effect transistors [94];
- photoinduced mechanical actuators [95];
- optical and thermal memory devices [96].

The key features used to compare the studies were the nature of input, nature of output, fabrication method, and technology readiness level. The nature of input and output describe the physical domains involved in the device operation. For sensors, the input is the sensed quantity and the output is the measured quantity. For actuators and switches, the input is the control quantity and the output quantity is the actuated or controlled quantity. 

Where the data were available, the input column also includes information about the specific quantity and the range studied in the paper, and the output column includes the specific quantity and either the sensitivity achieved or the range corresponding to the range of input. In some cases, the output was measured indirectly (for example, resistivity change measured using voltage), so the column includes both the quantity changing and the quantity measured.

The fabrication methods column briefly mentions the methods used to fabricate the VO_2_ structures and the type of structure fabricated for the particular device. Another key feature that was compared between the studies was the technology readiness level (TRL). The idea behind it is to show where a technology is in terms of development, with the lowest score corresponding to basic principles being observed and a theoretical application proposed, and the highest score corresponding to the technology being used commercially. While the assessment was inherently somewhat subjective, the scale used was loosely based on [97].

A score of 1 was given to applications that were proposed on the basis of a measurement of a physical property change in VO_2_. If a study also included more in-depth theoretical work related to the application, for example a simulation, a score of 2 was given. If the study included a fabricated device, the score was 3, and if it went beyond basic characterization of the device, for example, testing it in a specific application or a variety of different conditions, the score went up to 4. The highest score given in the comparison was 5, which went to the studies that demonstrated more robust prototypes with some level of reliability considerations.

The applications considered in Table 3 include applications for sensors and actuators. The sensors are mostly resistive, with a few examples of piezoelectric sensors. Also, mechanical actuators with electrical or optical inputs are being developed.

## 4. Discussion

Different applications of VO_2_ were considered in this review, with a focus on applications in sensors and actuators. Most of the sensors seem to be resistive in nature, relying on the resistivity change in VO_2_ under different changes in conditions; however, piezoelectric sensors have also been proposed. The most developed sensors that were looked at were resistive temperature sensors and resistive strain sensors. The idea of a mechanical microactuator is also being developed, as shown in multiple studies.

### 4.1. Applications: Further Discussion

The applications considered in this review include electrical, optical, thermal, mechanical, fluidic, and chemical domains. Also, some of the studies covered in this review have proposed dual-parameter sensors, which combine two sensors in one structure, such as a combined temperature and strain sensor.

#### 4.1.1. Resistive Strain Sensors

Different approaches to manufacturing a strain sensor using VO_2_ are being developed, with some of the studies showing a higher technology readiness level, where the manufactured prototypes are tested for repeatability and reliability, as well as applied to concrete applications, such as human health monitoring. Some of the devices are intended to be used as push switches, some as strain sensors, and others as combined temperature and strain sensors.

**Figure 9:**
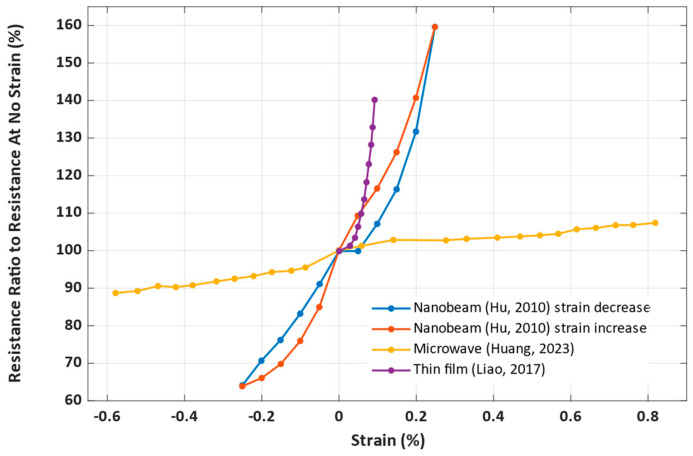
Sensitivity and measurement ranges of proposed strain sensors, based on (Hu, 2010) [73], (Huang, 2023) [74], and (Liao, 2017) [76].

The information from the studies that provided enough data to conclude the sensitivity characteristics of the sensors was compiled into the Figure 9, which shows the dependence between the resistance ratio (the resistance at a given strain divided by the resistance at no strain) and strain (the length of the sample divided by the length of the unstressed sample), both expressed in percentages.

The microwire sensor in [74] was tested for the highest bidirectional strain, −0.6% to 0.8%. Compared to other presented sensors, it has a good linearity, but a lower sensitivity. The thin film sensor in [75] has a higher sensitivity, but a poor linearity. The nanobeam sensor in [73] presents a sensor with both a good linearity and higher sensitivity, tested for strains −0.25% to 0.25%. The ink sensor in [77] was tested for strains from 0% to 3%, but the paper did not directly present data on the device’s sensitivity.

#### 4.1.2. Resistive Temperature Sensors

Temperature sensors have been developed using different manufacturing approaches, with the most used structures being films and powders. These applications show the greatest technology readiness levels among the applications considered. Some of the considered sensors are dual-parameter sensors, combining strain and temperature sensing into one device. Such devices are proposed especially for human monitoring and AI wearable applications, such as using a temperature sensor to access human body temperature and strain sensing to access pulse, facial expressions, hand gestures, etc.

The two more technologically ready sensors, [77,78], are produced by forming an ink as opposed to thin film deposition, with this fabrication approach being easier in the lab, making development quicker, but not necessarily ensuring further quick development as the technology moves towards scaling.

**Figure 10:**
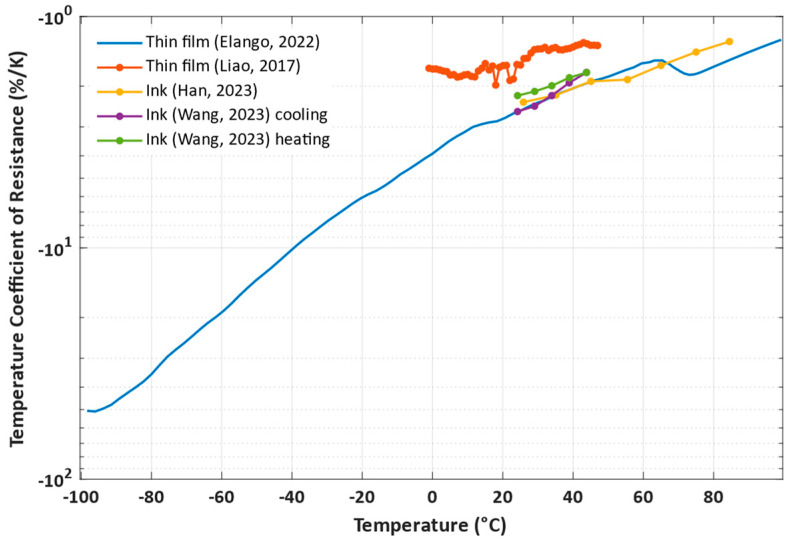
Sensitivity and measurement ranges of proposed temperature sensors, based on (Liao, 2017) [76], (Han, 2023) [77], (Wang, 2023) [78], and (Elango, 2022) [79].

Due to VO_2_ showing different properties before, during, and after phase transition, the operating range of a sensor can be defined to operate in one of the regions with a more linear characteristic or across multiple regions with a more complex characteristic. Figure 10 shows a summary of the proposed thermal sensors, their sensitivities, expressed as temperature coefficients of resistance, and their operating ranges. It is important to note that the temperature coefficients shown in Figure 10 somewhat differ from the values mentioned in the corresponding studies, as the coefficients were calculated for each of the points separately in order to show the temperature coefficient dependency on the operating temperature as opposed to obtaining an average value by calculating it from a linear fit of the data.

The considered temperature sensors operate in different temperature ranges, with many of the studies focusing on ranges appropriate for environmental and human-related temperature measurements. The best combination of linearity and high sensitivity is shown by [78], which operates in a narrow range of 25 °C to 45 °C. The greatest temperature range studied was −100 °C to 100 °C in [79], which also employed density functional theory computational studies and experimental measurements to determine the device’s performance under different humidity conditions. The study found that while there was a resistance change and temperature response rate change caused by humidity, by classifying the humidity conditions as dry, normal, and wet, the behaviour of the sensor could be relatively easily described in each of the regions.

#### 4.1.3. Resistive Gas Concentration Sensors

Resistive gas concentration sensors are being developed based on VO_2_ nanostructures (nanosheets and nanorods) and composite structures, which include materials such as Pd, WO_3_, Ag, and MoTe_2_. Experimental samples are being developed to detect different gases, such as NO_2_ in concentrations from 0.5 to 5 ppm, H_2_ in concentrations from 1 to 1000 ppm, and CH_4_ in concentrations from 500 to 4500 ppm [103,104,105,106,107].

#### 4.1.4. Mechanical Microactuators

Most of the microactuators considered in this review are microcantilever structures, with some kind of thin film stacking. Some of the works are based on VO_2_/Cr stacks, while others combine three to five layers. The most used method to deposit VO_2_ films was pulsed laser deposition. RF sputtering was also used. To pattern the films, most commonly, etching, and in one study, femtosecond laser direct writing were used. The proposed actuators switch between their undeformed and deformed states. While some works proposing continuous actuation as opposed to switching were found, it does not seem to be a viable approach without employing a feedback control system.

Many of the covered studies include multiple types of actuation, namely, directly thermal, optical with heating via laser, and electrical. For the purposes of this review, electrical actuation was considered. The electrical power applied to structures to ensure the switches was up to an order of magnitude different between the considered studies. Some studies, such as [83], showed similar deformations regardless of the actuation type. However, in others, for example in [80], the displacement due to electrical heating was almost ten times smaller than the displacement due to global heating. This hints to uniform power and heat distribution being a challenge for the electrical actuation of such actuators. However, many of the proposed designs tackled this challenge quite well. 

**Figure 11:**
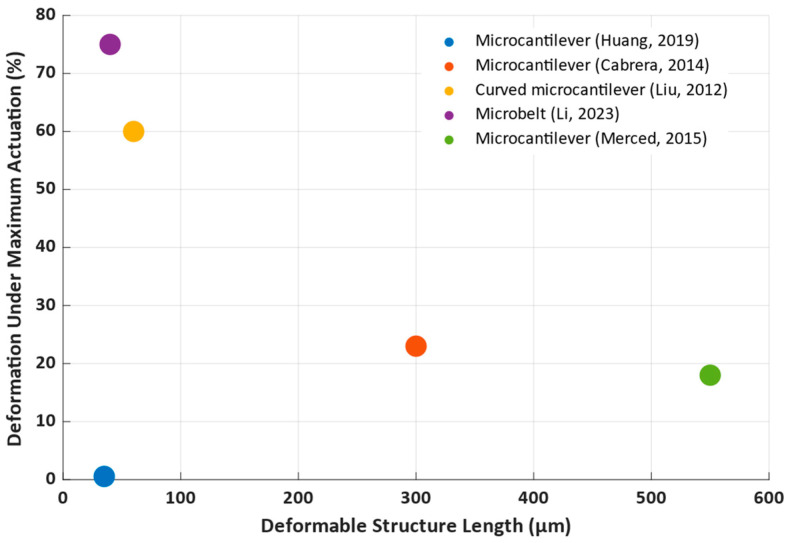
Relative deformation of the proposed microactuators and their sizes, based on (Huang, 2019) [80], (Cabrera, 2014) [81], (Liu, 2012) [82], (Li, 2023) [83], and (Merced, 2015) [84].

The sizes of the deforming parts of devices varied between the proposed solutions, with the smallest one being 35 µm long and the longest one being 550 µm. The output variable, mechanical displacement, was expressed as a percentage of the device length to allow for a comparison of the differently sized devices more fairly. However, care should be taken to not extrapolate the measured displacement/length ratio to different lengths, as the studies that compared multiple similar structures of different sizes showed different displacement/length ratios. The information about the sizes and displacements of the considered structures were compiled as Figure 11.

The smallest deformation among the considered studies was shown by [80], due to issues with uneven heating of the VO_2_ structure under electrically induced heating. Two longer devices showed a moderate displacement of 23% [81] and 18% [84]. Good performance was achieved in [84] by integrating a resistance sensor into the structure itself, which allowed for self-sensing of the actual actuator deformation and using a PID controller to more precisely control the actuator deflection. The highest deformation of 75% [83] and 60% [82] corresponded to the smaller devices. In both studies, the devices had a bent shape with a relatively high curvature and flattened, fully or partially, when voltage was applied.

Environmental conditions were also considered in some of the studies, with all actuators being tested in air, but some additionally in water and in a vacuum. Significant differences in actuation energy were observed for different environments, which is attributed mainly to different rates of heat exchange between the VO_2_ actuating layer and the environment.

#### 4.1.5. Other Applications

Other applications considered in this review are a resistive photodetector [98,99,100,101], piezoelectric strain sensor [102], and thermal fluid flow sensor [108,109]. While the piezoelectric effect was observed in VO_2_, the application as a strain sensor is only theoretically proposed. The application as a fluid sensor for microfluidics explores the thermally induced change in the resistivity of VO_2_ as applied to fluid flow measurement, with some sample devices being manufactured.

### 4.2. Performance Modification Methods

The methods of tuning the VO_2_ properties and phase change temperature observed in the reviewed studies include doping, using various nanostructures, such as microbelts, microwires, nanocrystals, nanosheets, etc., and synthesizing different types of composite structures, including thin film stacks and different combinations of nanoparticles.

### 4.3. Fabrication Methods and Structures

There does not seem to be a strong correlation between the type of sensor developed and the structure used, and different structures are being explored for different applications. Thin film structures are very popular for both actuators and different types of sensors. The methods for fabricating thin films are mostly different physical vapor deposition approaches, most notably pulsed laser deposition, DC sputtering, and RF sputtering.

Some applications use VO_2_ in ink formed by using a VO_2_ powder suspended in a liquid (example applications include photodetector, temperature, and strain sensors) [77,78,99]. This approach simplifies the manufacture of VO_2_ devices, at least at a laboratory test scale, and does not necessarily seem to yield devices with worse sensitivity values than other methods. Similarly, some gas sensors use drop and spin coating with a VO_2_ powder [104] or a nanorod solution [105].

Other structures explored include microbelts [83], microwires [74], micro- and nanocrystals [102], and single crystal nanobeams [73], for which both chemical and physical deposition methods are used. A microbelt structure, used as a mechanical microactuator, does not seem to lead to a superior sensitivity compared to thin film structures, as well as microwires, used in a resistive strain sensor application. Micro- and nanocrystals have been used as piezoelectric strain sensors, but the application of VO_2_ as a piezoelectric material does not seem to have been as actively explored as its application in resistive-type sensors and is in a rather theoretical state now. Single crystal nanobeams have been proposed for strain sensor applications and seem to provide a better sensitivity as compared to thin film counterparts.

Also, it has to be noted that the structures used in the studies with the highest technology readiness level were thin films, produced using DC sputtering [79,98], pulsed laser deposition [82], and polymer-assisted deposition [76], as well as ink-based designs [77,78]. 

## 5. Key Challenges

While VO_2_ is an exciting material with possible applications in many fields of technology, there are challenges related to its use in different applications.

### 5.1. Opportunities to Apply VO_2_ in Different Domains

The dependence between the temperature and resistivity of VO_2_ is one of the possible properties of the material that can be targeted to develop new devices; however, there are many other possible domains that can be affected by the change in properties or in which the phase transition can be triggered. This leaves an open opportunity to explore more ways to apply VO_2_ in different devices and fields. 

### 5.2. Performance-Related Challenges

A lot of applications of VO_2_ rely specifically on the IMT region. However, the temperature at which it happens, while reasonably close to room temperature, is still not practical for many applications and has to be modified using different approaches, which can for example, greatly increase hysteresis [22]. Furthermore, some applications rely on switching performance and some sensors require a linear characteristic change, further expanding the performance modification scope. 

### 5.3. Manufacturing Challenges 

There are different methods of manufacturing single-crystal and polycrystalline films and nanostructures. However, it is still challenging to precisely controlling the manufacture of VO_2_ nanostructures and achieving a high quality of films [36]. Currently, there is no simple, highly productive, and CMOS-compatible manufacturing approach for VO_2_ structures [71], and different approaches are being explored for manufacturing VO_2_-based devices.

### 5.4. Solutions 

Recent studies have shown that an intermediate metallic phase is also formed in VO_2_ thin films during the insulator–metal transformation due to the presence of donor impurities [110]. In addition, further metastable phases are also formed. The simultaneous presence of different phases can cause mechanical fatigue and malfunction in VO_2_-based micro- and nano-scale devices [70]. Further research is needed to address this problem.

To increase the commercialization of VO_2_ devices, it would be necessary to develop low-cost and high-volume manufacturing methods for VO_2_(M)-phase micro-/nanowires and nanostructures free of unwanted phases [65].

The size of the nanowire has a direct impact on the behavior of the VO_2_ IMT. Being able to precisely control the size and alignment of VO_2_ structures is a problem that needs solving [22].

In electronic devices, the IMT temperature of ~68 °C is often too high. This is currently reduced by the addition of dopants to the VO_2_, but the consequence is a slow transition rate and large hysteresis window [22]. There is a need to develop a method to reduce the IMT temperature without deteriorating these parameters. Furthermore, macroscopic defects (voids) cause local stresses and thus significantly affect the device performance. A possible solution exists by using a focused ion beam (FIB) and nanoindentation, such that the shape, location, and volume of macroscopic defects can be precisely controlled, allowing the hysteresis loop, IMT temperature, and conductivity to be tuned.

For VO_2_ in direct contact with air, device degradation may be caused by oxidation of V^4+^ ions to V^5+^ [70]. To solve this, the use of a passivation layer, encapsulation, and a VO_2_ core–shell structure can ensure better-quality VO_2_ films.

The hydrophobic HfO_2_ encapsulation structure can be used to prolong the lifetime of VO_2_ films, which also have excellent optical properties [111].

It is necessary to increase the stability of vanadium oxide devices against thermal, light, moisture, and oxygen environmental factors [20]. This requires a deeper understanding of their degradation mechanisms.

Vanadium oxides are toxic and can damage the liver, bones, and DNA of leukocytes, among other things [20]. Environmental impacts can be reduced by designing the manufacturing process and waste management appropriately. The encapsulation of the devices and application of silica coatings can seal vanadium from air and water.

The application of vanadium dioxide in IC devices is often limited by its thermal stability. VOx phase transition occurs at relatively low temperatures, which can be a significant bottleneck.

One of the methods that is used to modulate the critical temperature in vanadium dioxide was presented in [60], where it was shown how critical temperature could be adjusted through chemical doping, stoichiometric engineering, and advanced fabrication techniques.

Another vital approach is device structure optimization; the structure of the device using VOx should be optimized to reduce the thermal load on the material and improve the thermal dissipation characteristics of the device. At the system level, thermal management in the overall IC system is also crucial. This includes designing ICs with efficient heat dissipation paths, using materials with good thermal conductivity as heat spreaders, and managing the power consumption patterns of the ICs to minimize localized heating.

## 6. Conclusions

This paper highlights the evolving research on VO_2_, delving into its properties, phase transition mechanics, and potential applications. Studies on VO_2_, especially in the context of its insulator–metal transition and its resistivity change, have been ongoing since the 1960s. However, its relevance in the sensor and actuator field has surged in recent years, as evidenced by a significant increase in related publications and citations since 2010. According to the Dimensions database and Scopus, the annual output of VO_2_-focused studies in sensors and actuators has reached over 140 and 40, respectively, with citations exceeding 12,000 and 1000 in each database.

This review primarily focused on VO_2_’s applications in sensors and actuators. Most sensors based on VO_2_ are resistive, building on its change in resistivity under varying conditions. However, piezoelectric sensors are also emerging. The most advanced of these are resistive temperature and strain sensors. Additionally, the development of mechanical microactuators using VO_2_ is gaining more attention.

Despite these advancements, there is a need for further research to develop simpler, more robust, and reliable VO_2_-based sensors and actuators. The sustained interest in VO_2_ for various applications is evident, with ongoing research exploring both new uses and enhancements to existing designs. The scope of this research spans from theoretical materials science studies for a deeper understanding of VO_2_ to practical aspects such as simplifying device fabrication for mass production.

## Figures and Tables

**Table 1:** Some physical properties of VO_2_(R) and VO_2_(M).

Physical Property	VO_2_(R)	VO_2_(M)
Crystal system	Tetragonal	Monoclinic
Resistivity	2 × 10^−4^ to 5 × 10^−4^ Ω·cm	10^−3^ to 10 Ω·cm
Seebeck coefficient	−23 to −21 μV/°C	−30 to −1000 μV/°C
Mass magnetic susceptibility	8 × 10^−6^ A·m^2^/kg	1 × 10^−6^ A·m^2^/kg
Infrared optical transmittance *	55 to 75%	75 to 90%
Volumetric thermal expansion coefficient	37.2 × 10^−6^ K^−1^	20.3 × 10^−6^ K^−1^
Thermal conductivity	400 to 700 W/m·°C	400 to 700 W/m·°C

* 780 to 2500 nm.

**Table 2:** Most cited publications on the topic.

Publication Name	Year	Citation Count	Reference
Femtosecond Structural Dynamics in VO_2_ during an Ultrafast Solid–Solid Phase Transition	2001	1535	[55]
Oxide Electronics Utilizing Ultrafast Metal–Insulator Transitions	2011	1051	[56]
Evidence for a structurally-driven insulator-to-metal transition in VO_2_: A view from the ultrafast timescale	2004	848	[57]
VO_2_: Peierls or Mott–Hubbard? A view from band theory	1994	827	[41]
Dynamical Singlets and Correlation-Assisted Peierls Transition in VO_2_	2005	815	[48]

**Table 3:** Review of recent papers concerning VO_2_ applications in the sensor and actuator fields.

Application	Nature of Input	Nature of Output	Fabrication Method and Structure	TRL	Ref.
Resistive photodetector	Light, wavelength of 365 to 850 nm	Electrical, resistivity, current of 273 to 2067 mA/W	Pulsed direct current sputtering (thin film)	4	[98]
	Light, wavelength of 400 nm to 20 µm	Electrical, resistivity, current of 70 to 268 mA/W	Ink was formed by mixing IPA/2-butanol solution with VO_2_ powder	3	[99]
	Light, wavelength of 650 to 980 nm	Electrical, resistivity, current of 78 to 353 mA/W	Pulsed laser deposition (thin film)	3	[100]
	Light, wavelength of 350 to 950 nm	Electrical, resistivity, current of 40 to 1100 mA/W	Hydrothermal approach (thin film)	3	[101]
Resistive strain sensor	Mechanical, strain measurement, 0% to 3% deformation	Electrical, resistivity, current change ratio of 1.2	Ink was formed by mixing latex and VO_2_ powder	5	[77]
	Mechanical, strain measurement, 0% to 0.1% deformation	Electrical, resistivity, resistance change of 40%	Polymer-assisted deposition (thin film)	4	[76]
	Mechanical, strain measurement, −0.25% to 0.25% deformation	Electrical, resistivity, 50 MΩ to 120 MΩ	Physical vapour deposition (single crystal nanobeams)	3	[73]
	Mechanical, strain measurement, −0.6% to 0.8% deformation	Electrical, resistivity, at 0.1 V, current of 26.2 nA to 31.7 nA	Chemical vapour deposition (microwire)	3	[74]
	Mechanical, strain switching	Electrical, resistivity, 20 kΩ to 5 MΩ	RF sputtering (thin film)	3	[75]
Piezoelectric strain sensor	Mechanical, strain measurement	Electrical, piezoelectric, d33 = 12 pm/V	Vapor transport (micro- and nanocrystals)	1	[102]
Resistive H_2_ concentration sensor	Chemical, H_2_ gas concentration, 1 to 1000 ppm	Electrical, resistivity, 25 MΩ to 0.2 MΩ	Radio frequency-assisted oxide molecular beam epitaxy (thin film)	3	[103]
Resistive CH_4_ concentration sensor	Chemical, CH_4_ gas concentration, 500 to 4500 ppm	Electrical, resistivity, 2.5 MΩ to 3 MΩ	Hydrothermal and drop coating method (powder coating)	3	[104]
Resistive temperature sensor	Thermal, temperature, 25 °C to 45 °C	Electrical, resistivity, −2.71%/K	Ink was formed by mixing poly(3,4-ethylenedioxythiophene):poly(styrene sulfonate) and VO_2_ powder	5	[78]
	Thermal, temperature, 25 °C to 80 °C	Electrical, resistivity, −0.9%/K	Ink was formed by mixing latex and VO_2_ powder	5	[77]
	Thermal, temperature, −3 °C to 47 °C	Electrical, resistivity, −1.12%/K	Polymer-assisted deposition (thin film)	4	[76]
	Thermal, temperature, −100 °C to 100 °C	Electrical, resistivity, −2.43%/K	DC sputtering (thin film)	4	[79]
Resistive NO_2_ concentration sensor	Chemical, NO_2_ gas concentration, 1 to 5 ppm	Electrical, resistivity, 2.5 kΩ to 4.5 kΩ	Hydrothermal method and spin coating (nanorod coating)	3	[105]
	Chemical, NO_2_ gas concentration, 0.5 to 5 ppm	Electrical resistivity, 11 kΩ to 8 kΩ	Hydrothermal method (vertical nanosheet array)	3	[106]
	Chemical, NO_2_ gas concentration, 1 to 5 ppm	Electrical resistivity, 55 MΩ to 70 MΩ	Chemical vapour deposition (nanorods)	3	[107]
Mechanical microactuator	Electrical, 0 to 1.6 mW or light, 0 to 4 mW	Mechanical, displacement, 36 µm, 60% of length	Pulsed laser deposition (thin film)	4	[82]
	Electrical, 0 to 120 mW or light, 808 nm or laser, 0 to 250 mW	Mechanical, displacement, 30 µm for electrical actuation, 25 µm for laser, 75% and 63% of length	Femtosecond laser direct writing (microbelt)	3	[83]
	Electrical, current, 2 to 5 mA	Mechanical, displacement, 100 µm, 18% of length	Pulsed laser deposition (thin film)	3	[84]
	Electrical, current, 0 to 9.8 mA	Mechanical, displacement, 73.5 µm to 142.2 µm deformation, 23% of length	Pulsed laser deposition (thin film)	3	[55]
	Electrical, voltage, 0 to 5 V	Mechanical, displacement, 0.22 µm, 0.55% of length	RF magnetron sputtering (thin film)	3	[80]
Thermal fluid flow sensor	Fluidic, flow rate, 0 to 37.8 μL/s	Electrical, resistivity, resistance change from 2.2 kΩ to 3.5 kΩ	Pulsed laser deposition (thin film)	3	[108]
	Fluidic, flow rate, 0 to 0.2 μL/min	Electrical, resistivity, voltage of 1.34 V/(μL/min)	Pulsed laser deposition (thin film)	2	[109]

## Data Availability

The data presented in this study are available upon request from the corresponding author.
